# Management of children with fever and neutropenia: results of a survey in 51 pediatric cancer centers in Germany, Austria, and Switzerland

**DOI:** 10.1007/s15010-020-01462-z

**Published:** 2020-06-10

**Authors:** Max Scheler, Thomas Lehrnbecher, Andreas H. Groll, Ruth Volland, Hans-Jürgen Laws, Roland A. Ammann, Philipp Agyeman, Andishe Attarbaschi, Margaux Lux, Arne Simon

**Affiliations:** 1 Department of Pediatric Oncology and Hematology, Medical Faculty, University Children’s Hospital of Cologne, Cologne, Germany; 2 Pediatric Hematology and Oncology, Hospital for Children and Adolescents, University Hospital, Goethe University Frankfurt am Main, Frankfurt, Germany; 3 Department of Pediatric Oncology, Hematology and Clinical Immunology, University Hospital Düsseldorf, Düsseldorf, Germany; 4 Infectious Disease Research Program, Center for Bone Marrow Transplantation and Department of Pediatric Hematology/Oncology, University Children’s Hospital Münster, Münster, Germany; 5 Division of Pediatric Hematology/Oncology, Department of Pediatrics, Inselspital, Bern University Hospital, University of Bern, Bern, Switzerland; 6 Pediatric Hematology and Oncology, St. Anna Children’s Hospital, Medical University of Vienna, Vienna, Austria; 7 Pediatric Oncology and Hematology, Childrens’ Hospital Medical Center, Saarland University Clinic, Kirrberger Str. Building 09, 66421 Homburg, Germany

**Keywords:** Pediatric cancer patients, Febrile neutropenia, Survey, Antimicrobial treatment

## Abstract

**Purpose:**

Investigation of the current practice of diagnostics and treatment in pediatric cancer patients with febrile neutropenia.

**Methods:**

On behalf of the German Society for Pediatric Oncology and Hematology and the German Society for Pediatric Infectious Diseases, an Internet-based survey was conducted in 2016 concerning the management of febrile neutropenia in pediatric oncology centers (POC). This survey accompanied the release of the corresponding German guideline to document current practice before its implementation in clinical practice.

**Results:**

In total, 51 POCs participated (response rate 73%; 43 from Germany, and 4 each from Austria and Switzerland). Identified targets for antimicrobial stewardship concerned blood culture diagnostics, documentation of the time to antibiotics, the use of empirical combination therapy, drug monitoring of aminoglycosides, the time to escalation in patients with persisting fever, minimal duration of IV treatment, sequential oral treatment in patients with persisting neutropenia, indication for and choice of empirical antifungal treatment, and the local availability of a pediatric infectious diseases consultation service.

**Conclusion:**

This survey provides useful information for local antibiotic stewardship teams to improve the current practice referring to the corresponding national and international guidelines.

**Electronic supplementary material:**

The online version of this article (10.1007/s15010-020-01462-z) contains supplementary material, which is available to authorized users.

## Introduction

Due to their underlying disease and anticancer treatment, pediatric oncology patients are at an increased risk of infectious complications. Fever during neutropenia may be the only sign of an infectious episode, but in the majority of pediatric cancer patients with fever during neutropenia, the focus of the underlying infection remains unclear and cannot be documented microbiologically (e.g., bloodstream infection with a pathogen isolated) or clinically (e.g., pneumonia). Because of the risk of a complicated clinical course and the spectrum of pathogens causing severe and life-threatening bacterial infections in pediatric cancer patients, timely inpatient treatment with broad-spectrum antibiotics is the standard of care [1, 2].

Despite this consensus, previous surveys performed in pediatric cancer centers from different countries revealed a high level of heterogeneity in many key topics of clinical management [3–8]. In May 2016, the working group for infectious complications in the immunocompromised child of the German Society for Pediatric Oncology and Hematology (GPOH) and the German Society for Pediatric Infectious Diseases (DGPI) released consensus recommendations [9], concerning the diagnostics and treatment of fever without a focus on neutropenic pediatric cancer patients (FN), excluding hematopoietic stem cell transplant recipients and children and adolescents with clinical signs of sepsis, septic shock or infection-related organ failure. The aim of the present survey was to explore the institutional standards of treatment of FN in pediatric oncology centers (POCs) from Germany, Austria, and Switzerland, to compare the current clinical practice with the recommendations of the new guideline [9] and to identify targets for improvement from the perspective of antimicrobial stewardship.

## Methods

Referring to the recent German FN guidelines [9], the authors developed an Internet-based anonymized survey (Survey Monkey™; San Mateo, USA). The main topics and detailed questions were finalized after repeated rounds of internal discussion in the GPOH/DGPI working group (Supplemental Table 1 contains the questions and the corresponding information from the guideline). The survey did not include clinical case vignettes [8] and did not collect original local standard operation procedure documents [4]. It is important to emphasize that (by definition) the FN population comprised by the German guideline does not include children and adolescents with clinical signs of sepsis, septic shock or infection-related organ failure. In total, 70 pediatric oncology centers (POCs) were contacted by e-mail and asked for participation. Typically, the head of the department or a responsible senior pediatric oncology consultant was contacted. One reminder was sent out to hospitals which did not reply to the first e-mail. The survey was released on March 14, 2016, briefly before publication of the guideline [9], and closed on July 27, 2016, i.e., 2 months after the publication. The POCs were arbitrarily categorized into three different sizes, namely small (≤ 40 newly diagnosed patients/year), medium (41–75), and large (> 75) centers. In addition, we also analyzed whether the affiliation (university hospital vs. academic tertiary care children hospital) affected the results.

Standard statistical methods (SPSS Version 24 IBM SPSS Statistics) were used to analyze potential correlations of the results with the size of the participating POCs. Datasets derived from the online protected database were checked for duplicates from the same center. Fisher’s test was used to examine differences between categorical variables to a significance level of 5% (*p* < 0.05). Since the survey did not contain individual patient data, participation was voluntary and the participating oncologist consented to the anonymous cumulative analysis; an approval by ethics committee was not necessary.

## Results

### Number and characteristics of participating institutions and internal guideline

Fifty-one pediatric oncologists from 51 GPOH-affiliated POCs participated (response rate 73%; 43 from Germany, and 4 each from Austria and Switzerland, respectively); however, not all of them answered all questions completely. Therefore, we provide the corresponding number of POCs, which have answered to a particular question, in parenthesis (e.g., 10/51). Sixty-five percent of all 51 POCs were university hospitals and 35% were academic tertiary care pediatric hospitals. The median number of inpatient pediatric oncology beds was 14 (interquartile range; IQR 9–18; min. 4, max. 30). In 2015, the median number of newly admitted patients (de novo and relapsed malignancies) was 50 (IQR 33–80; min. 15, max. 170). Ninety percent of the participating POCs had an internal standard operation procedure document detailing the routine management of patients with FN. Tables 1, 2, and 3 show the analysis of the corresponding results referring to small, medium, and large centers as well as to the affiliation (university vs. academic tertiary care children hospital).

**Table 1:** Comparison of the statistical significance (Fisher’s exact test) concerning the type of pediatric oncology centers (POCs): university hospital vs. academic tertiary pediatric care facility

Questions from the survey	Answer options	University hospitals (*n* = 33)	Academic tertiary pediatric care facility (*n* = 18)	Statistical significance (Fisher’s exact test, *p* values)
Standard blood cultures (1) Which blood culture vials are utilized?	a) Only aerobic culture vial	4	2	0.472
	b) Aerobic and anaerobic culture vial	28	15	
	c) Additional mycosis culture vial	0	1	
Standard blood cultures (2) From which access is the blood sample for the cultures taken from?	a) Only from the Broviac/Port-A-Cath	31	18	0.534
	b) From the Broviac/Port-A-Cath and peripheral venous	2	0	
	c) Only peripheral venous	0	0	
Standard blood cultures (3) Which volume is taken from a child with a body weight of 15 kg?	a) 1–3 ml per vial (z.B. Bactec® Paeds)	11	8	0.073
	b) 3–5 ml per vial	16	8	
	c) 5–10 ml per vial	6	2	
Laboratory tests on admission to the hospital—blood count?	a) Yes	33	18	Ø
	b) No	0	0	
Laboratory tests on admission to the hospital—CRP?	a) Yes	33	18	Ø
	b) No	0	0	
Laboratory tests on admission to the hospital—interleukin 8?	a) Yes	0	0	Ø
	b) No	33	18	
Laboratory tests on admission to the hospital—interleukin 6?	a) Yes	2	0	0.534
	b) No	31	18	
Laboratory tests on admission to the hospital—procalcitonin?	a) Yes	5	6	0.164
	b) No	28	12	
Laboratory tests on admission to the hospital—liver function tests?	a) Yes	30	18	0.544
	b) No	3	0	
Laboratory tests on admission to the hospital—creatinine?	a) Yes	32	18	1.000
	b) No	1	0	
Laboratory tests on admission to the hospital—coagulation tests?	a) Yes	13	10	0.378
	b) No	20	8	
Laboratory tests on admission to the hospital—blood gas analysis?	a) Yes	19	11	1.000
	b) No	14	7	
Is a urine sample analyzed at all times?	a) Yes	20	17	**0.010**
	b) No	13	1	
“Time to antibiotics (TTA)” Is the exact period of time between the time point of admission to the hospital and the first dose of antibiotics documented?	a) Yes	15	10	0.551
	b) No	18	7	
First-line antibiotic treatment regime: for a pediatric cancer patient with fever in neutropenia without a focus we generally use:	a) An empiric monotherapy	17	9	1.000
	b) An empiric combination therapy	16	8	
Does there exist a fixed rule when to add an antimycotic agent for patients at high risk for an invasive fungal infection with persistent fever without a focus?	a) After 72 h	12	8	0.922
	b) After 96 h	9	4	
	c) According to individual decisions	8	4	
	d) According to own specifications	4	1	
Is the antibiotic treatment generally switched in clinically stable patients with persistent fever? If the answer is yes, at which time point does this switch take place?	a) After 48 h	16	8	1.000
	b) After 72 h	10	5	
	c) According to individual decisions	7	4	
How long is the minimum duration of iv antibiotic treatment in pediatric cancer patients with FN (good clinical condition, sterile initial blood cultures and no fever or at least 24 h)?	a) < 48 h	2	0	0.271
	b) 48 h	10	2	
	c) 72 h	14	8	
	d) > 72 h	7	7	
Do you usually stop iv antibiotic treatment despite the occurrence of leukocyte recovery?	a) Yes	21	8	0.366
	b) No, recovery of leukocytes required	12	9	
Do patients receive an oral antibiotic continuation treatment whose fever has resolved but who are still neutropenic?	a) Yes	6	3	0.908
	b) No	7	2	
	c) In particular cases	20	12	
Are there regular interdisciplinary conferences of pediatric hematologists/oncologists and microbiologists held to analyze data on invasive pathogens and their in vitro sensitivity profile for a defined retrospective period of time (e.g., every 6 or 12 months)?	a) Yes	14	8	0.773
	b) No	19	9	
Does a regular clinical visit with a pediatric infectious disease specialist (or optionally with a clinical microbiologist) take place in your pediatric oncology center?	a) Yes	18	5	0.136
	b) No	15	12	

Bold value indicates significant results (*p* < 0.05)

**Table 2:** Comparison of the statistical significance (Fisher’s exact test) concerning the center size: “small vs. medium large vs. large” pediatric oncology centers (POCs)

Questions from the survey	Answer options	“Small” POCs (*n* = 21)	“Medium large” POCs (*n* = 15)	“Large” POCs (*n* = 15)	Statistical significance (Fisher’s exact test, *p* values)
Standard blood cultures (1) Which blood culture vials are utilized?	a) Only aerobic culture vial	2	1	3	0.631
	b) Aerobic and anaerobic culture vial	18	14	11	
	c) Additional mycosis culture vial	1	0	0	
Standard blood cultures (2) From which access is the blood sample for the cultures taken from?	a) Only from the Broviac/Port-A-Cath	20	14	15	1.000
	b) From the Broviac/Port-A-Cath and peripheral venous	1	1	0	
	c) Only peripheral venous	0	0	0	
Standard blood cultures (3) Which volume is taken from a child with a body weight of 15 kg?	a) 1–3 ml per vial (z.B. Bactec® Paeds)	9	5	5	0.073
	b) 3–5 ml per vial	12	7	5	
	c) 5–10 ml per vial	0	3	5	
Laboratory tests on admission to the hospital—blood count?	a) Yes	21	15	15	Ø
	b) No	0	0	0	
Laboratory tests on admission to the hospital—CRP?	a) Yes	21	15	15	Ø
	b) No	0	0	0	
Laboratory tests on admission to the hospital—interleukin 8?	a) Yes	0	0	0	Ø
	b) No	21	15	15	
Laboratory tests on admission to the hospital—interleukin 6?	a) Yes	1	0	1	1.000
	b) No	20	15	14	
Laboratory tests on admission to the hospital—procalcitonin?	a) Yes	5	2	4	0.755
	b) No	16	13	11	
Laboratory tests on admission to the hospital—liver function tests?	a) Yes	20	15	13	0.471
	b) No	1	0	2	
Laboratory tests on admission to the hospital—creatinine?	a) Yes	21	15	14	0.588
	b) No	0	0	1	
Laboratory tests on admission to the hospital—coagulation tests?	a) Yes	5	10	8	**0.030**
	b) No	16	5	7	
Laboratory tests on admission to the hospital—blood gas analysis?	a) Yes	10	9	11	0.376
	b) No	11	6	4	
Is a urine sample analyzed at all times?	a) Yes	17	12	8	0.180
	b) No	4	3	7	
“Time to antibiotics (TTA)” Is the exact period of time between the time point of admission to the hospital and the first dose of antibiotics documented?	a) Yes	10	10	5	0.209
	b) No	10	5	10	
Firs-line antibiotic treatment regime: for a pediatric cancer patient with fever in neutropenia without a focus we generally use:	a) An empiric monotherapy	8	8	10	0.291
	b) An empiric combination therapy	12	7	5	
Does there exist a fixed rule when to add an antimycotic agent for patients on high risk for an invasive fungal infection with persistent fever without a focus?	a) After 72 h	7	7	6	0.687
	b) After 96 h	5	3	5	
	c) According to individual decisions	4	4	4	
	d) According to own specifications	4	1	0	
Is the antibiotic treatment generally switched in clinically stable patients with persistent fever? If the answer is yes, at which time point does this switch take place?	a) After 48 h	9	8	7	0.927
	b) After 72 h	6	5	4	
	c) According to individual decisions	5	2	4	
How long is the minimum duration of iv antibiotic treatment in pediatric cancer patients with FN (good clinical condition, sterile initial blood cultures and no fever or at least 24 h)?	a) < 48 h	1	0	1	0.765
	b) 48 h	5	3	4	
	c) 72 h	8	6	8	
	d) > 72 h	6	6	2	
Do you usually stop iv antibiotic treatment despite the occurrence of leukocyte recovery?	a) Yes	10	10	9	0.673
	b) No, recovery of leukocytes required	10	5	6	
Do patients receive an oral antibiotic continuation treatment whose fever has resolved but who are still neutropenic?	a) Yes	4	4	1	0.705
	b) No	4	2	3	
	c) In particular cases	12	9	11	
Are there regular interdisciplinary conferences of pediatric hematologists/oncologists and microbiologists held to analyze data on invasive pathogens and their in vitro sensitivity profile for a defined retrospective period of time (e.g., every 6 or 12 months)?	a) Yes	5	6	11	**0.020**
	b) No	15	9	4	
Does a regular clinical visit with a pediatric infectious diseases specialist (or optionally with a clinical microbiologist) take place in your pediatric oncology center?	a) Yes	8	5	10	0.152
	b) No	12	10	5	

Bold values indicate significant results (*p* < 0.05)

**Table 3:** Interdisciplinary conferences concerning antibiograms of invasive pathogens and infectious disease consultations

Question 1	Answer options	Question 2	Answer options	Correlation (Fisher’s exact test, *p*-value)
Are there regular interdisciplinary conferences of pediatric hematologists/oncologists and microbiologists held to analyze data on invasive pathogens and their in vitro sensitivity profile for a defined retrospective period of time (e.g., every 6 or 12 months)?	Yes (*n* = 22)	First-line antibiotic treatment regime: for a pediatric cancer patient with fever in neutropenia without a focus we generally use:	An empiric monotherapy (*n* = 13)	0.407
			An empiric combination therapy (*n* = 9)	
	No (*n* = 28)		An empiric monotherapy (*n* = 13)	
			An empiric combination therapy (*n* = 15)	
Does a regular clinical visit with a pediatric infectious diseases specialist (or optionally with a clinical microbiologist) take place in your pediatric oncology center?	Yes (*n* = 23)		An empiric monotherapy (*n* = 16)	**0.027**
			An empiric combination therapy (*n* = 7)	
	No (*n* = 27)		An empiric monotherapy (*n* = 10)	
			An empiric combination therapy (*n* = 17)	

Bold value indicates significant results (*p* < 0.05)

### Fever criteria and methods for measuring temperature

**Fig. 1:**
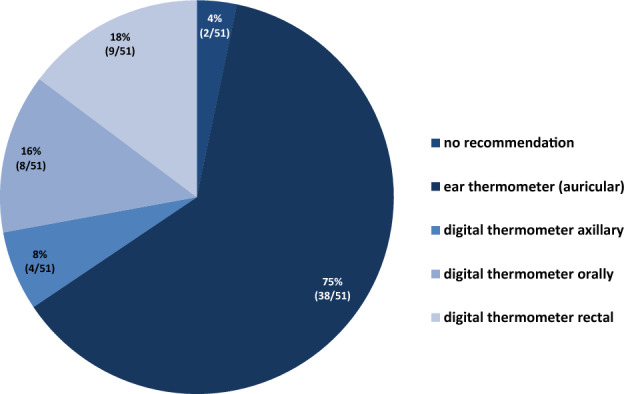
Recommended method of temperature measurement (inpatient setting)

The survey offered a choice of three different definitions of fever, but participants could also enter free-text comments. Overall, 78% (40/51) of the POCs use the definition “temperature once > 38.5 °C or > 38 °C with repeated measurement after one hour”; 2% (*n* = 1) use “once > 39 °C” and 6% (*n* = 3) once > 38 °C. Concerning the method of temperature measurement, we asked about the current standards in inpatients (Fig. 1). Remarkably, 41% of the POCs did not have a defined standard method for temperature measurement in outpatients (data not shown).

### Vital signs on admission

Vital signs documented by all centers (*n* = 51) in a patient admitted with FN are temperature (100%), heart rate (100%), blood pressure (100%), and actual body weight (98%). With lower frequencies, the POCs document respiratory rate in 55% (28/51), oxygen saturation at room air (pulse oximetry) in 75% (38/51), and median arterial pressure in 78% (40/51; RR_diast_ plus 1/3 of the difference RR_syst_ − RR_diast_).

### Blood cultures

**Fig. 2:**
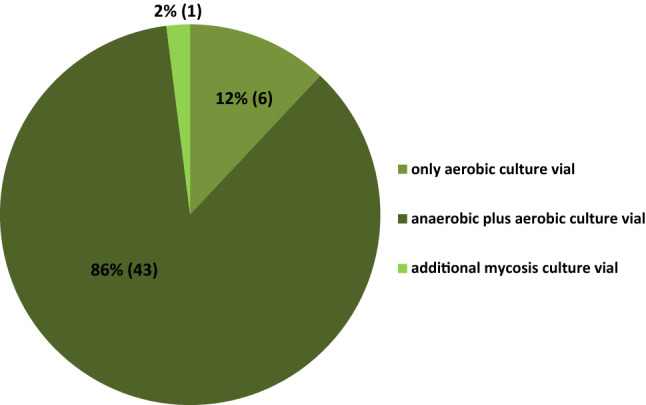
Which blood culture vials are utilized?

**Fig. 3:**
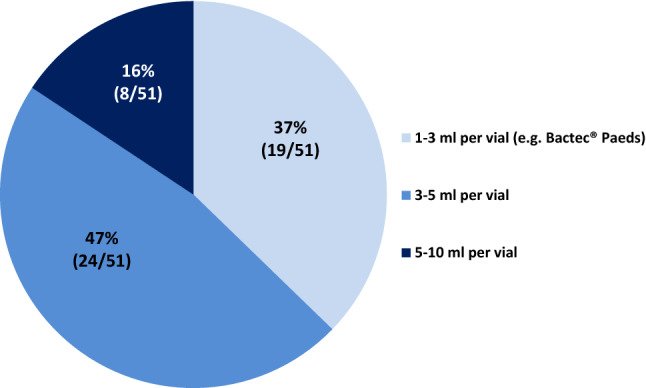
Which minimal volume is sampled in a patient with FN and a body weight of 15 kg?

Nearly all POCs (96%; 49/51) sample blood cultures only from the central venous access device (if present), whereas only two (4%) regularly also collect blood cultures from a peripheral vein. Figures 2 and 3 show further details of the local standard of blood culture diagnostics.

Only two centers (4%; 10/51) regularly draw blood cultures 24 h after the onset of antibiotic treatment, 20% (10/50) of the centers draw cultures once daily in patients with ongoing fever despite antibiotic treatment, and 46% (23/50) of the institutions draw repeat blood cultures only in patients with persistent fever prior to the escalation of empiric antibiotic treatment (e.g., after 48–96 h).

### Further laboratory diagnostics

On admission of a patient with FN, all centers order a peripheral blood cell count (including a differential WBC), a serum C-reactive protein, creatinine, and transaminases (ALT, AST). Additional surrogate parameters indicating systemic inflammation and infection such as interleukin 6, interleukin 8, and procalcitonin are regularly determined in none, 4% (2/51), and 22% (11/51) of all centers. 73% (37/51) perform a urine dipstick test with or without a urine culture in case of positive findings. This practice was significantly seen more often in academic teaching hospitals compared to university hospitals [94% (17/18) vs. 61% (20/33); *p* = 0.01; Table 1]. Thirty-one (59%; 31/51) stated to perform a venous blood gas analysis on admission; details (e.g., concerning the availability of lactate levels) were not asked for.

### Availability of PCR based viral diagnostics

**Fig. 4:**
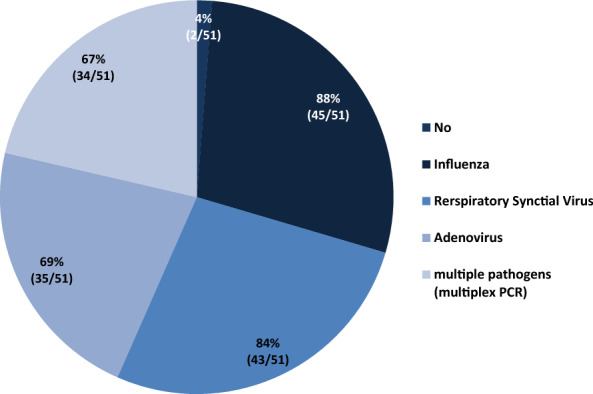
Availability of (rt)PCR-based methods to detect viral pathogens in respiratory secretions

Only 2 of 51 POCs (4%) did not have the availability to detect viral pathogens in respiratory secretions by (rt)PCR methods (Fig. 4). Sixty-seven percent of all POCs are capable of performing multiplex PCRs to detect a defined panel of respiratory viral pathogens.

### Time to antibiotics

The lag time or the interval from the time of admission to the time of the first antibiotic administration is documented regularly in 50% (25/50) of all POCs, without significant difference between small, medium, and large centers [48% (10/21) vs. 67% (10/15) vs. 33% (5/15), respectively].

### First-line antibiotic therapy

Half of all centers (52%; 26/50) use antibacterial monotherapy as first-line treatment of neutropenic pediatric oncology patients with fever without a focus, and 48% (24/50) use an empirical combination therapy. The proportion of small size centers using combination therapy (80%) is larger than the corresponding proportion in median (47%) and large centers (33%; difference statistically not significant).

The preferred first-line betalactam antibiotic in most POCS (61%; 30/49) is piperacillin–tazobactam, followed by ceftazidime (24%; 12/49), cefepime and ceftriaxone (4%; 2/49) each, the latter in combination with amikacin). Only three POCs (6%) use imipenem/cilastatin or meropenem as first-line treatment. Forty-four of 49 POCs (86%) provided specific information regarding the use of aminoglycosides (AGL) combination treatment; of those, 55% (24/49) prefer tobramycin, 34% (15/49) gentamicin, and 11% (5/49) amikacin. Nearly all POCs (94%; 45/48) administer AGLs once daily. Of 48 POCs responding to this question, 71% (*N* = 34) assess AGL trough levels even in patients with normal serum creatinine, whereas 21% (10/48) do not measure AGL trough levels regularly. In contrast, AGL peak levels (*C*_max_) are determined in only 8% (4/48). The exact time point of trough level sampling differed substantially between the POCs (data not shown).

### Time to escalation in patients with persisting fever

In patients with persisting FN, who are in stable clinical condition without microbiologically or clinically defined infection, 48% of POCs (24/50) change (escalate) the initial empirical antibacterial regimen after 48 h, 30% (15/50) after 72 h, and 22% (11/50) after clinical reassessment on an individual case to case basis.

### Minimum duration of IV antibiotic treatment

In patients, who are in a good clinical condition with negative initial blood cultures and no fever in the last 24 h, two POCs (4%; 2/50) state to execute a minimal IV treatment duration below 48 h. The minimum duration of IV treatment is 48 h in 24% (12/50), and 72 h in 44% (22/50). Twenty-eight percent (14/50) of all POCs treat these patients for more than 72 h. University hospitals have a shorter minimal treatment duration than academic teaching hospitals [> 72 h in 21% (7/33) vs. 42% (7/18 = 42%); difference not significant].

Concerning the recovery of leukocyte counts, more than half of the POCs (58%; 29/50) do not require the recovery of the leukocytes (e.g., neutrophils > 0.5 × 10/9/L) for the decision to stop the IV ABT, whereas 42% (21/50) of all POCs do not stop ABT prior to signs of leukocyte recovery. Eighty-six percent of all POCs discharge the patient on the same day on which antibacterial treatment is discontinued.

In patients, in whom the IV ABT is stopped despite persistent neutropenia, 18% (9/50) never continue with oral antibiotics, 64% (32/50) of all POCs continue treatment with an oral antibiotic in selected cases, and 18% (9/50) regularly continue with sequential oral therapy. In the nine centers, which stated to use sequential oral therapy in patients with persisting neutropenia, eight different antibiotics (antibiotic combinations) are utilized including ciprofloxacin in four centers.

### Empirical antifungal treatment

Twenty of 50 POCs (40%) start empirical antifungal treatment (AFT) in patients at high risk of invasive fungal infection (IFI) after 72 h of fever unresponsive to broad-spectrum antibiotics, whereas 26% (13/50) start AFT after 96 h; 24% (12/50) decide on the indication on an individual case-to-case basis. The preferred first-line antifungals for empirical AFT in FN patients are (multiple answers allowed): liposomal amphotericin B (AmBisome™) 84% (43/51), caspofungin 16% (8/51), and voriconazole 14% (7/51). In one POC each, the attending physicians recommend micafungin or conventional amphotericin B.

### Interdisciplinary conferences and infectious disease consultation

Regular interdisciplinary conferences of pediatric hematologists/oncologists and microbiologists to analyze data on invasive pathogens and their in vitro sensitivity profile take place in 46% (23/50) of all POCs. Regarding center size, large centers perform these meetings in 73% (11/15), small centers in 25% (5/21) and median-sized centers in 40% (6/15; *p* = 0.20; Table 2). The proportion of POCs using first-line monotherapy is lower in centers, which regularly organize such an interdisciplinary conference [41% (13/28) vs. 54% (13/21); *p* = 0.41]. Of all POCs (n = 50), 54% (*n* = 27) do not have the opportunity to consult a pediatric infectious disease specialist at their institution. The availability of a pediatric infectious disease consultation service is greater in large centers [67% (10/15) vs. 40% (8/20) in small POCs; *p* = 0.152) and in university hospitals relative to academic teaching hospitals [55%; (18/33) vs. 29% (5/17); *p* = 0.136]. POCs in which a pediatric infective disease consultation is performed regularly, less often use empiric first-line combination therapy is used [30% (7/23) vs. 63% (17/27); *p* = 0.027; Table 3).

## Discussion

This multicenter survey describes the current clinical practice of the management of FN without a clinically or microbiologically defined focus on POCs in Germany, Austria, and Switzerland. The response rate (73%) is in the upper range of comparable surveys from other countries [4, 8, 10].

Most POCs in Germany, Austria, and Switzerland are affiliated at University hospitals or academic tertiary care pediatric facilities, which reflects the current situation in these countries with few small- and large-, but many medium-size POCs. Since our survey was performed in parallel to the release of the 2016 German AWMF guideline [9], the results depict the situation before the main components and recommendations of this guideline could be implemented. However, it is important to note that the international guidelines on FN in the pediatric setting have been available starting in 2012 (updated in 2017 [11]. The German guideline differs only in a few issues from these international guidelines [12], including that the German guideline is not applicable to patients, who need intensive care due to severe sepsis, it does not recommend a priori risk stratification in high- and low-risk groups, and it argues against the use of meropenem (a carbapenem) as first-line treatment in stable patients. One important purpose of this survey was to identify potential targets for antibiotic stewardship (ABS).

More than 90% of all participating POCs have a defined internal standard operation procedure concerning the management of FN. Most POCs used the same definition for fever and neutropenia [13, 14]. Remarkably, 41% of the participating POCs do not recommend one defined standard method for temperature measurement in outpatients. The definition of fever and the method of temperature measurement should be harmonized, also to enable comparison of results between POCs [15]. Otherwise, a specific patient may or may not be hospitalized and treated with IV antibiotics only depending on the POC he attends to [3]. In pediatric oncology patients with FN, the timely detection of those patients with severe sepsis is of utmost importance, since clinical management in case of severe sepsis clearly differs from patients who only have FN [16]. Most POCS follow the German guidelines [9] and draw at least one blood culture set from the central venous access device (CVAD) before they start empiric antibiotic treatment (eABT). In our survey, only half of all POCs (47%) recognize that there is a minimal blood culture volume required for in a child with a bodyweight of 15 kg (at least 5 ml per bottle) [17, 18], whereas some use bottles provided for culturing 1–3 ml of blood from neonates and infants < 10 kg in all patients. This practice may significantly reduce blood culture sensitivity. The question, whether more than at least one blood culture set drawn on day 1 and on day 2 of treatment is of any benefit for the clinical management, remains a matter of debate [19, 20]. Rosenblum et al. analyzed 220 FN episodes in 105 patients and identified a pathogen in 11% of repeated cultures drawn in those patients with persistent fever [21]. If the initial blood culture has yielded a pathogen, control cultures are mandatory to guide further therapy and to confirm that a single positive culture yielding, e.g., coagulase-negative staphylococci is not a contamination.

The heterogeneous utilization of different biomarkers in our survey reflects that in febrile neutropenic patients without a focus, no biomarker is capable to definitely confirm or exclude a bacterial infection and to predict the clinical course [22]. Relying on experience (not on study results), most POCs use the C-reactive protein to confirm the response to antibiotic treatment. In some patients, leukocyte recovery is accompanied by a second increase in CRP values.

Minimizing the time to antibiotics (TTA) has been defined as an important goal of treatment; there seems to be an association between longer TTA and impaired safety [23]. Half of all POCs regularly document the TTA. In general, the GPOH guideline [9] recommends a first-line monotherapy with piperacillin–tazobactam, ceftazidime or cefepime (the latter two agents not in patients with severe mucositis). In contrast to the international guidelines [11, 24], the German guideline strictly argues against the empirical use of carbapenems in the first-line treatment of pediatric FN to avoid the selection of carbapenem-resistant pathogens. Empirical use of meropenem is recommended in case of clinical signs of a severe infection or sepsis, and may be considered in patients colonized (or previously infected) with ESBL-producing bacteria [25–27].

Keeping in mind the very low incidence of bloodstream infections due to ESBL-producing *Gram-*negative *Enterobacteriacerales* in recent studies from Germany, Switzerland, and the Netherlands [28, 29]*,* the reason for the high percentage of POCs using initial combination therapy (48%) remains unclear. In this respect, national [9] and international guidelines [11] may help to promote substantial changes in clinical management concerning initial monotherapy [30]. Other surveys and multicenter evaluations addressed this issue too [3, 31]. In Switzerland, the use of ceftriaxone plus amikacin adds antipseudomonal activity to ceftriaxone [32].

In cases with AGL combination treatment, most POCs use tobramycin or gentamicin as short time infusion once daily. Nowadays, this mode of administration represents the state of the art [33, 34]. Interestingly, only 8% of all POCs determine the Cmax (target: 8–10 × MIC; e.g., 10–20 mg/L, referring to an MIC of 2 µg/ml) to evaluate the appropriateness of individual AGL dosing. In addition, out of all POCs, 20% do not regularly determine AGL trough levels in patients with normal serum creatinine, although many of these patients may have received other potentially nephrotoxic or ototoxic treatments (e.g., platinum derivatives, furosemide, irradiation, etc.). This topic of supportive care would probably benefit from a less heterogeneous approach.

The GPOH guideline recommends escalating the empiric ABT in stable patients with persistent fever after 72 h if the patient shows neutropenia without signs of recovery [9]. This cutoff (72 h) has been established in many studies investigating the use of different empirical antibiotics [30]. There is no data confirming the benefit of changing the eABT earlier in a stable patient (e.g., after 48 h, as done by 48% of the POCs). Some POCs (22%) decide about this question on an individual basis. This may include the decision not to change the initial eABT in a stable patient whose neutrophils are expected to recover in the next few days [11]. Watchful waiting at least 72 h or even longer in this situation may reduce the cumulative consumption of second- and third-line ABT.

Irrespective of hematological recovery, nearly two-thirds of all POCs (58%) stop antibiotics in a stable patient with negative cultures, who is afebrile for 24 h, and has no severe mucositis or clinical signs of a focal infection. The GPOH guideline [9] clearly supports this approach, but argues against the oral continuation of eABT in most cases.

In neutropenic patients, many POCs (68%) discharge the patient but continue treatment with oral antibiotics. Remarkably, those centers mentioned eight different oral antibiotics/antibiotic combinations, including fluoroquinolones. This heterogeneity clearly reflects a lack of evidence, since the risk of secondary infection, in particular with *P. aeruginosa* (fluoroquinolones) [35] in patients eligible for oral sequence treatment is very low [36, 37].

About one-third of all POCs continue IV treatment for more than 72 h. This may negatively affect the quality of life of the patients and their families and increases the risk of nosocomial infections [38]. Concerning empirical antifungal treatment (eAFT), the guideline defines certain groups with high risk of invasive fungal infection (IFI) [39]. The guideline suggests using liposomal amphotericin B or caspofungin after 96 h of fever despite eABT [9], and provides details on diagnostic tools to confirm or exclude an IFI, as much as possible. Notably, 14% of all POCs use voriconazole for eAFT, although this drug is not licensed for eAFT and needs therapeutic drug monitoring [40].

The final questions of the survey tried to elucidate the access to some core elements of Antibiotic Stewardship (ABS) programs in this particular setting, more precisely.

- Interdisciplinary conferences to discuss the POC’s cumulative antibiogram (e.g., pathogens detected in blood cultures and their in vitro sensitivity) with clinicians, microbiologists, infectious disease specialists, and IPC personnel.
- A prospective audit and feedback service provided by pediatric infectious disease (PID) specialists from an ABS team [41].

Such interdisciplinary conferences take place in only 46% of all POCs. In addition, only 46% of all POCs have access to PID consultations, which reflects the profound shortage of PID specialists in pediatric health-care facilities in Germany. Interestingly, university centers more often have PID services, and POCs with PID support less often use first-line combination therapy in pediatric patients with FN. Hopefully, the emerging threat of infections due to multidrug-resistant pathogens and adverse short- and long-term effects of inadequate ABT will lead to the implementation of ABS programs in all POCs in the near future.

## Electronic supplementary material

Below is the link to the electronic supplementary material.

Supplementary file 1 (DOCX 32 kb)

## Data Availability

Provided on request to the corresponding author.

## References

[1] Lehrnbecher T (2019). Treatment of fever in neutropenia in pediatric oncology patients. Curr Opin Pediatr.

[2] Af-Sandeberg M, Johansson E, Wettergren L, Bjork O, Hertting O, Nilsson A (2017). Antibiotic use during infectious episodes in the first 6 months of anticancer treatment-A Swedish cohort study of children aged 7–16 years. Pediatr Blood Cancer.

[3] Bate J, Gibson F, Selwood K, Skinner R, Phillips B, Chisholm JC (2013). A reaudit of current febrile neutropenia practice in UK paediatric oncology centres prior to implementation of NICE guidance. Arch Dis Child.

[4] Delebarre M, Tiphaine A, Martinot A, Dubos F (2016). Risk-stratification management of febrile neutropenia in pediatric hematology-oncology patients: results of a French nationwide survey. Pediatr Blood Cancer.

[5] Fisher BT, Gerber JS, Leckerman KH, Seif AE, Huang YS, Li Y (2013). Variation in hospital antibiotic prescribing practices for children with acute lymphoblastic leukemia. Leuk Lymphoma.

[6] Cox A, Bradford N (2014). Management of febrile neutropenia in pediatric oncology across Queensland, Australia: a retrospective review on variations between locations. J Pediatr Oncol Nurs.

[7] Maxwell RR, Egan-Sherry D, Gill JB, Roth ME (2017). Management of chemotherapy-induced febrile neutropenia in pediatric oncology patients: A North American survey of pediatric hematology/oncology and pediatric infectious disease physicians. Pediatr Blood Cancer.

[8] Haeusler GM, Slavin MA, Bryant PA, Babl FE, Mechinaud F, Thursky KA (2018). Management of fever and neutropenia in children with cancer: a survey of Australian and New Zealand practice. J Paediatr Child Health.

[9] Deutsche Gesellschaft für pädiatrische Infektiologie (DGPI), Gesellschaft für Pädiatrische Onkologie und Hämatologie (GPOH). Diagnostik und Therapie bei Kindern mit onkologischer Grunderkrankung, Fieber und Granulozytopenie (mit febriler Neutropenie) außerhalb der allogenen Stammzelltransplantation. Arbeitsgemeinschaft Wissenschaftlicher Fachgesellschaften (AWMF). 2016;Registernummer 048/14(Finale Version 23.01.2016).

[10] Livadiotti S, Milano GM, Serra A, Folgori L, Jenkner A, Castagnola E (2012). A survey on hematology-oncology pediatric AIEOP centers: prophylaxis, empirical therapy and nursing prevention procedures of infectious complications. Haematologica.

[11] Lehrnbecher T, Robinson P, Fisher B, Alexander S, Ammann RA, Beauchemin M (2017). Guideline for the management of fever and neutropenia in children with cancer and hematopoietic stem-cell transplantation recipients: 2017 update. J Clin Oncol.

[12] Lehrnbecher T, Groll A, Agyeman P, Ammann RA, Attarbaschi A, Behrends U (2018). Recommendations for diagnostics and therapy of children with cancer presenting with fever and neutropenia—comparison of two current guidelines. Klin Padiatr.

[13] Haeusler GM, Phillips RS, Lehrnbecher T, Sung L, Ammann RA (2013). The reporting of outcomes in studies of fever and neutropenia in children with cancer: time for consensus. Pediatr Blood Cancer.

[14] Haeusler GM, Phillips RS, Lehrnbecher T, Thursky KA, Sung L, Ammann RA (2015). Core outcomes and definitions for pediatric fever and neutropenia research: a consensus statement from an international panel. Pediatr Blood Cancer.

[15] Binz P, Bodmer N, Leibundgut K, Teuffel O, Niggli FK, Ammann RA (2013). Different fever definitions and the rate of fever and neutropenia diagnosed in children with cancer: a retrospective two-center cohort study. Pediatr Blood Cancer.

[16] Barking C, Masjosthusmann K, Rellensmann G, Ehlert K, Zollner S, Jocham S (2020). Treatment of children with cancer and/or hematopoietic stem cell transplantation in the intensive care unit: experience at a large European pediatric cancer center. J Pediatr Hematol Oncol.

[17] Gaur AH, Giannini MA, Flynn PM, Boudreaux JW, Mestemacher MA, Shenep JL (2003). Optimizing blood culture practices in pediatric immunocompromised patients: evaluation of media types and blood culture volume. Pediatr Infect Dis J.

[18] Dien Bard J, McElvania TE (2016). Diagnosis of bloodstream infections in children. J Clin Microbiol.

[19] Petty LA, Sokol EA, Bartlett AH, McNeer JL, Alexander KA, Pisano J (2016). Repeated blood cultures in pediatric febrile neutropenia: would following the guidelines alter the outcome?. Pediatr Blood Cancer.

[20] Neemann K, Yonts AB, Qiu F, Simonsen K, Lowas S, Freifeld A (2016). Blood cultures for persistent fever in neutropenic pediatric patients are of low diagnostic yield. J Pediatr Infect Dis Soc.

[21] Rosenblum J, Lin J, Kim M, Levy AS (2013). Repeating blood cultures in neutropenic children with persistent fevers when the initial blood culture is negative. Pediatr Blood Cancer.

[22] Phillips RS, Sung L, Amman RA, Riley RD, Castagnola E, Haeusler GM (2016). Predicting microbiologically defined infection in febrile neutropenic episodes in children: global individual participant data multivariable meta-analysis. Br J Cancer.

[23] Koenig C, Schneider C, Morgan JE, Ammann RA, Sung L, Phillips B (2020). Association of time to antibiotics and clinical outcomes in patients with fever and neutropenia during chemotherapy for cancer: a systematic review. Support Care Cancer.

[24] Lehrnbecher T, Phillips R, Alexander S, Alvaro F, Carlesse F, Fisher B (2012). Guideline for the management of fever and neutropenia in children with cancer and/or undergoing hematopoietic stem-cell transplantation. J Clin Oncol.

[25] Averbuch D, Avaky C, Harit M, Stepensky P, Fried I, Ben-Ami T (2017). Non-fermentative Gram-negative rods bacteremia in children with cancer: a 14-year single-center experience. Infection.

[26] Averbuch D, Cordonnier C, Livermore DM, Mikulska M, Orasch C, Viscoli C (2013). Targeted therapy against multi-resistant bacteria in leukemic and hematopoietic stem cell transplant recipients: guidelines of the 4th European Conference on Infections in Leukemia (ECIL-4, 2011). Haematologica.

[27] Tamma PD, Han JH, Rock C, Harris AD, Lautenbach E, Hsu AJ (2015). Carbapenem therapy is associated with improved survival compared with piperacillin-tazobactam for patients with extended-spectrum beta-lactamase bacteremia. Clin Infect Dis.

[28] Ammann RA, Laws HJ, Schrey D, Ehlert K, Moser O, Dilloo D (2015). Bloodstream infection in paediatric cancer centres-leukaemia and relapsed malignancies are independent risk factors. Eur J Pediatr.

[29] Miedema KG, Winter RH, Ammann RA, Droz S, Spanjaard L, de Bont ES (2013). Bacteria causing bacteremia in pediatric cancer patients presenting with febrile neutropenia–species distribution and susceptibility patterns. Support Care Cancer.

[30] Robinson PD, Lehrnbecher T, Phillips R, Dupuis LL, Sung L (2016). Strategies for empiric management of pediatric fever and neutropenia in patients with cancer and hematopoietic stem-cell transplantation recipients: a systematic review of randomized trials. J Clin Oncol.

[31] Herd F, Bate J, Chisholm J, Johnson E, Phillips B (2016). Variation in practice remains in the UK management of paediatric febrile neutropenia. Arch Dis Child.

[32] Pereira CA, Petrilli AS, Carlesse FA, Luisi FA, da Silva KV, de Martino Lee ML (2009). Cefepime monotherapy is as effective as ceftriaxone plus amikacin in pediatric patients with cancer and high-risk febrile neutropenia in a randomized comparison. J Microbiol Immunol Infect.

[33] Germovsek E, Barker CI, Sharland M (2017). What do I need to know about aminoglycoside antibiotics?. Arch Dis Child Educ Pract Ed.

[34] Houot M, Pilmis B, Thepot-Seegers V, Suard C, Potier C, Postaire M (2016). Aminoglycoside use in a pediatric hospital: there is room for improvement-a before/after study. Eur J Pediatr.

[35] Sung L, Manji A, Beyene J, Dupuis LL, Alexander S, Phillips R (2011). Fluoroquinolones in children with fever and neutropenia: a systematic review of prospective trials. Pediatr Infect Dis J.

[36] Manji A, Beyene J, Dupuis LL, Phillips R, Lehrnbecher T, Sung L (2012). Outpatient and oral antibiotic management of low-risk febrile neutropenia are effective in children–a systematic review of prospective trials. Support Care Cancer.

[37] Morgan JE, Cleminson J, Atkin K, Stewart LA, Phillips RS (2016). Systematic review of reduced therapy regimens for children with low risk febrile neutropenia. Support Care Cancer.

[38] Teuffel O, Ethier MC, Alibhai SM, Beyene J, Sung L (2011). Outpatient management of cancer patients with febrile neutropenia: a systematic review and meta-analysis. Ann Oncol.

[39] Groll AH, Castagnola E, Cesaro S, Dalle JH, Engelhard D, Hope W (2014). Fourth European Conference on Infections in Leukaemia (ECIL-4): guidelines for diagnosis, prevention, and treatment of invasive fungal diseases in paediatric patients with cancer or allogeneic haemopoietic stem-cell transplantation. Lancet Oncol.

[40] Kadam RS, Van Den Anker JN (2016). Pediatric clinical pharmacology of voriconazole: role of pharmacokinetic/pharmacodynamic modeling in pharmacotherapy. Clin Pharmacokinet.

[41] Mehta JM, Haynes K, Wileyto EP, Gerber JS, Timko DR, Morgan SC (2014). Comparison of prior authorization and prospective audit with feedback for antimicrobial stewardship. Infect Control Hosp Epidemiol.

