# Supplementary material for: Management of children with fever and neutropenia: results of a survey in 51 pediatric cancer centers in Germany, Austria, and Switzerland
Source: Infection. 2020 Jun 10;48(4):607–18. doi: 10.1007/s15010-020-01462-z (PMC7395019; doi:10.1007/s15010-020-01462-z)
Supplement: Supplementary file 1 — (DOCX 32 kb) [file 15010_2020_1462_MOESM1_ESM.docx]

| **Questions from the survey** | **Answer options from the survey** | **Corresponding statements from the GPOH/DGPI consensus recommendations** |
| --- | --- | --- |
| 1. Type of pediatric oncology center (POC)? | 🞎 University hospital  🞎 Academic tertiary pediatric care hospital or regional pediatric care facility | ∅ |
| 1. Number of inpatient pediatric oncology beds? | Number | ∅ |
| 1. Number of newly admitted patients per year (first diagnosis or relapse)? | Number | ∅ |
| 1. Does your facility have a written standard operation procedure for the diagnostics and treatment of neutropenic fever without a focus (FN) in pediatric cancer patients? | 🞎 Yes  🞎 No | The implementation of a standard operation procedure (set out in writing) for the diagnostics and treatment of fever without a focus in pediatric cancer patients with FN is highly recommended. In order to analyze and – if indicated – improve the fixed treatment strategies continuously in everyday clinical practice, the above mentioned implementation should be accompanied by practical training for all involved employees and appropriate measures of quality control (“plan-do-check-act-cycles”). |
| 1. Which fever criteria are used? | 🞎 > 38,5°C  🞎 > 38 °C (repeated measurements)  🞎 > 39°C  🞎 Further criteria (free text) | A body temperature above 38.5°C (or a persistent body temperature above 38.0°C in repeated measurements) is considered as fever. |
| 1. How is the body temperature measured (outpatient setting)?   Multiple replies possible! | 🞎 No recommendation  🞎 Recommendation “orally”  🞎 Recommendation “axillary”  🞎 Recommendation “rectal” | The body temperature of pediatric cancer patients with neutropenia should not be measured rectally due to the increased risk of mucosal injuries which could finally serve as a portal of entry for pathogens. Since there do exist quite different methods of temperature measurement it is recommended to **specify the one method that should be used in the inpatient and the outpatient setting**. The patients and their parents should be informed about the recommended method. Ideally, the body temperature measurement is then performed uniformly. |
| 1. How is the body temperature measured (inpatient setting)?   Multiple replies possible! | 🞎 No recommendation  🞎 Ear thermometer (auricular)  🞎 Digital thermometer axillary  🞎 Digital thermometer orally  🞎 Digital thermometer rectal |  |
| 1. Which vital signs are measured and documented on admission to the hospital?   Multiple replies possible! | 🞎 Body temperature  🞎 Heart rate  🞎 Blood pressure (systolic/diastolic)  🞎 Blood pressure (mean arterial pressure,  MAP)  🞎 Respiratory rate  🞎 Oxygen saturation (pulse oximetry)  🞎 Body weight | All of the items that are listed here are recommended to be measured and documented on hospital admission. |
| 1. Standard blood cultures (1)   Which blood culture vials are utilized? | 🞎 Only aerobic culture vial  🞎 Aerobic and anaerobic culture vial  🞎 Additional mycosis culture vial | It is generally recommended to utilize a set of blood culture vials (aerobic and anerobic). For children with a body weight less than 10 kg a specialized so called “Paeds vial” (e.g. the BD Bactec®) should be used. These vials are not recommended for children with a body weight > 10kg because the lower culture volume may decreases the sensitivity of pathogen detection. The utilization of an additional mycosis culture vial is not generally recommended. It can be useful in patients with higher risk for invasive fungal infections or with proven invasive candida infection in another compartment (e.g. hepato-lienal foci or candida esophagitis). |
| 1. Standard blood cultures (2)   From which access is the blood sample for the cultures taken from? | 🞎 Only from the Broviac / Port-A-Cath  🞎 From the Broviac / Port-A-Cath and  from a peripheral vein  🞎 Only from a peripheral vein | In case the patient has a continuous central venous access device (CVAD), a set of blood culture vials (aerobic and anaerobic) should be drawn from every lumen of the CVAD since any of them could be the source of bacteremia. After thorough evaluation, the expert panel suggests not to draw an additional peripheral venous blood culture sample. |
| 1. Standard blood cultures (3)   Which volume is taken from a child with a body weight of 15 kg? | 🞎 1-3 ml per vial (z.B. Bactec® Paeds)  🞎 3-5 ml per vial  🞎 5-10 ml per vial | The consensus statements recommend standardized blood sample volumes according to the patient’s body weight:   - < 10 kg: 1-3 ml (“Paeds vial”) or 5 ml (aerobic vial) - < 10 kg - 20 kg: 2 x 5 ml (aerobic and anaerobic vial) - > 20 kg: 2 x 10 ml (aerobic and anaerobic vial) |
| 1. Which laboratory tests are performed on admission to the hospital?   Multiple replies possible! | 🞎 Blood count and differential blood count  🞎 C-reactive protein (CRP)  🞎 Interleukin 8 (IL-8)  🞎 Interleukin 6 (IL-6)  🞎 Procalcitonin (PCT)  🞎 Liver function tests  🞎 Creatinine  🞎 Coagulation tests  🞎 Blood gas analysis (venous or capillary) | Recommendation:  The following laboratory tests should be performed regularly on admission to the hospital:   - Blood cell count and differential blood cell count (absolute neutrophil count?) - Sodium, potassium, creatinine, ALT - CRP or PCT or IL-6 or IL-8 (according to local convention) - In case of reduced general condition and/or dehydration or persistent vomiting: venous or capillary blood gas analysis (electrolytes, blood glucose, base excess, lactate) - Optionally: Coagulation tests according to a locally fixed standard (e.g., in patients with ALL after asparaginase treatment). |
| 1. Do you perform a urine sample analysis (dip stick)? | 🞎 Yes  🞎 No | Because of the following reasons the examination of a urine sample is not recommended for all patients:   - To receive a conclusive urine specimen (sufficient sensitive and specific) a sample of fresh midstream urine or the use of a sterile disposable catheter is needed. Both procedures need time and are not really suitable for smaller children. - Bag urine is unsuitable for culture, because contamination frequently causes false-positive findings - Leukocyturia as a common sign for urinary tract infection often fails in children with severe neutropenia - The patients often receive the first dose of antibiotics before a urine sample can be obtained |
| 1. “Time to Antibiotics (TTA)”   Is the exact period of time between the time point of admission to the hospital and the first dose of antibiotics documented? | 🞎 Yes  🞎 No | The first dose of antibiotics after admission to the hospital of a pediatric cancer patients with fever and neutropenia should be administered as quickly as possible. Preferably, the interval between the admission to the hospital and the first administration of i.v. antibiotics should not exceed 60 minutes. All critical time points (time of admission, time of first administration of antibiotics) should be documented in the patient’s record. |
| 1. Are (rt)PCR based methods to detect viral pathogens in respiratory secretion available in your hospital?   Multiple replies possible! | 🞎 No  🞎 Influenza  🞎 Respiratory Syncytial Virus  🞎 Adenovirus  🞎 Multiple pathogenes (multiplex PCR) | If there are signs for a viral infection in the patient’s history or the clinical examination targeted virological diagnostics are recommended. In several clinical trials, more than one third of all fever episodes in pediatric cancer patients were due to viral infections and the majority of these infections were caused by respiratory viruses. Therefore early viral diagnostics might help to recognize severe or life-threatening viral infections and – in case of a patient in good clinical condition – the detection of a respiratory viral infection might enable the physician to stop or at least not to escalate the primary antibiotic treatment. However this question remains unsolved at this time. |
| 1. First-line antibiotic treatment regime:   For a pediatric cancer patient with fever in neutropenia without a focus we generally use: | 🞎 An empiric monotherapy.  🞎 An empiric combination therapy. | The recommended first-line antibiotic treatment for pediatric cancer patients with fever in neutropenia without a focus is an empiric monotherapy with an appropriate broad-spectrum antibiotic agent (e.g. Piperacillin-Tazobactam, Ceftazidime or cefepime). |
| 1. Beta-lactam antibiotic of choice for first-line treatment in pediatric cancer patients with FN? | 🞎 Piperacillin-Tazobactam  🞎 Ceftazidime  🞎 Cefepime  🞎 Ceftriaxone without an aminoglycoside  🞎 Ceftriaxone plus an aminoglycoside  🞎 Imipenem/Cilastatin or Meropenem | The following substances are basically suitable for the empiric antibiotic first-line treatment of pediatric cancer patients with FN:   - Piperacillin-Tazobactam - Ceftazidime or Cefepime - Meropenem or Imipenem/Cilastatin   In general, we recommend the use of Piperacillin-Tazobactam since it showed equal efficacy compared to 3^rd^ or 4^th^ generation cephalosporins and carbapenems (or a combination therapy with aminoglycosides).  Basically, Ceftazidime and Cefepime are equally effective alternatives to Piperacillin-Tazobactam, however Ceftazidime should not be used in patients with AML and high-grade mucositis because of its reduced efficacy for viridans streptococci. |
| 1. If you use an aminoglycoside for initial combination therapy, which one do you prefer? | 🞎 Gentamicin  🞎 Tobramycin  🞎 Amikacin | An initial combination therapy with an aminoglycoside is not generally recommended.  In the following situations or selected patients an initial combination therapy with an aminoglycoside should be considered:   - Patients with acute lymphoblastic leukemia (ALL) and high-dose corticosteroid therapy within the induction and/or re-induction therapy, after receiving high-risk treatment cycles or within the framework of recurrent leukemia - Patients with transient arterial hypotension and oliguria that responds sustainably to a bolus infusion of 40 ml crystalloid solution per kg body weight - Patients with an increased risk for translocation of gram-negative bacteria due to high grade mucositis of the bowel and/or perianal border disorders.   The guideline does not contain any preferences, concerning the choice of a specific aminoglycoside. |
| 1. If you use an aminoglycoside for an initial combination therapy, how often it is administered? | 🞎 Three times a day  🞎 Twice daily  🞎 Once daily | It is recommended to administer all aminoglycosides once daily. A therapeutic drug monitoring according to the AWMF-Guideline  „Sepsis in children after the neonatal period“ (AWMF Registry number 024/025) is highly recommended.  No further details concerning this drug monitoring are provided. |
| 1. If you use an aminoglycoside for an initial combination therapy, do you perform a therapeutic drug monitoring (considering patients with normal serum creatinine)? | 🞎 Trough level  🞎 Peak level  🞎 No therapeutic drug monitoring |  |
| 1. At which timepoints is the therapeutic drug monitoring performed? | 🞎 Trough level (free text)  🞎 Peak level (free text) |  |
| 1. Do you extract additional blood cultures during the course of hospitalization? | 🞎 Once after 24 hours  🞎 Daily in the case of persistent fever  🞎 Only prior to the change of antibiotic  treatment  🞎 Further criteria (free text) | In the case of persistent fever in neutropenia a new set of blood cultures should be drawn from the CVAD prior to any change of the antibiotic treatment regimen. There is no data supporting the sampling of additional blood cultures at other time points in children with initially negative blood cultures, therefore it is not mandatory. |
| 1. Does there exist a fixed rule when to add an antifungal agent for patients on high risk for an invasive fungal infection with persistent fever without a focus? | 🞎 After 72 hours  🞎 After 96 hours  🞎 According to individual decisions | For patients with high risk for invasive fungal infection^[[1]](#footnote-1)^ and persistent fever despite an adequate empiric antibacterial treatment, empiric antifungal therapy should be started after 96 hours and should be continued until the neutrophils recover > 0.5 x 10^9^/L. |
| 1. Which antifungal agent do you generally use for the empiric first-line treatment in pediatric cancer patients with FN? | 🞎 Fluconazole  🞎 Amphotericin B (conventional)  🞎 Liposomal Amphotericin B (AmBisome®)  🞎 Caspofungin  🞎 Micafungin  🞎 Voriconazole | Recommended options for the antifungal empiric first-line treatment in alphabetical order (officially licensed drugs for this special indication):   - Caspofungin (day 1: 70 mg/m^2^, followed by 50 mg/m^2^/d, max. 70 mg/d) - liposomal Amphotericin B (3 mg/kg/d) |
| 1. Is the antibiotic treatment generally switched in clinically stable patients with persistent fever? If the answer is yes, at which time point does this switch regularly takes place? | 🞎 After 48 hours  🞎 After 72 hours  🞎 According to individual decisions | There do not exist any significant prospective randomized controlled clinical trials that deal with the issue of the best possible escalation therapy in pediatric cancer patients with FN and persistent fever. Because of that lack of evidence explicit recommendations cannot be given. Relying on clinical experiences and expert opinion, a few basic statements can be made:   - The well-known limit of 72 hours when talking about “persistent fever” has evolved historically. Eventually it is an arbitrary convention. Nevertheless, the majority of patients with FN remain afebrile after that time interval - Fever as the only symptom in a clinically stable patient with neutropenia in good general condition is an inadequate criterion to assess the efficacy of antibiotic treatment. Escalation of antibiotic treatment in such a situation is generally not necessary if the expected regeneration of the leukocyte count is right around the corner (just a few days). - The antibiotic agent which is used for an therapeutic escalation should have a broader antibacterial spectrum than the first-line treatment (e.g. Ceftazidime replaced by Piperacillin-Tazobactam or Piperacillin-Tazobactam replaced by Meropenem) |
| 1. How long is the minimum duration of iv antibiotic treatment in pediatric cancer patients with FN (good clinical condition, sterile initial blood cultures and no fever or at least 24 hours)? | 🞎 < 48 hours  🞎 48 hours  🞎 72 hours  🞎 > 72 hours | The recommended minimum duration of iv antibiotic treatment in patients eligible for this guideline is 72 hours. |
| 1. Do you usually stop iv antibiotic treatment despite there is no sign of leukocyte recovery? | 🞎 Yes  🞎 No, recovery of leukocytes required | Whether the current leukocyte count is considered as a criterion to stop or continue IV antibiotic treatment, should be decided on an individual case basis after a thorough evaluation by the responsible pediatric oncologist. Here, in particular, the remission status of the underlying disease (especially in leukemia) and the expected duration of leukopenia play a crucial role. |
| 1. Are the patients whose iv antibiotic treatment was stopped allowed to leave the hospital on the same day (if there are no other reasons that require a continuation of the hospitalization)? | 🞎 Yes  🞎 No, not earlier than the following day | After the IV antibiotic treatment was stopped there is no reason to continue hospitalization from an infectious disease perspective (there may be other reasons). |
| 1. Do patients whose fever has resolved but who are still neutropenic receive an oral antibiotic as sequential therapy? | 🞎 Yes  If yes: with which drug: (free text)  🞎 No  🞎 In particular cases | In general, sequential oral treatment with antibiotics is not recommended after a minimum of 72 hours IV treatment. |
| 1. Do you use adjuvant antimicrobial lock solutions for pediatric cancer patients with FN and a CVAD (in addition to the IV antibiotic treatment)? | 🞎 No  🞎 Yes: Ethanol  🞎 Yes: Taurolidine  🞎 Yes: Antibiotic-block ± heparin | The guideline does not comment on this issue. |
| 1. Are there regular interdisciplinary conferences of pediatric hematologists/oncologists and microbiologists held to analyze data on invasive pathogens and their in vitro sensitivity profile for a defined retrospective period of time (e.g. every 6 or 12 months)? | 🞎 Yes  🞎 No | Since the department of pediatric hematology/oncology can be regarded as a department responsible for patients with high risk for severe invasive bacterial infections, the department of microbiology (the attending laboratory) should provide data on invasive pathogens (species) and their in vitro sensitivity (antibiogram) for a defined retrospective period of time. Ideally this data is provided in an interdisciplinary conference where upcoming questions and contentious issues can be discussed. The provided data at best match to a so called “actionable feedback” since they may impact on decisions concerning the empiric antibiotic treatment standard. |
| 1. Does a regular clinical visit with a pediatric infectious diseases specialist (or optionally with a clinical microbiologist) take place in your pediatric oncology center? | 🞎 Yes  🞎 No | The guideline does not comment on this issue |

1. AML induction therapy, relapsed leukemia not in remission, severe neutropenia for at least 10 days, early phase after stem cell transplantation, server Graft versus Host Disease. [↑](#footnote-ref-1)
